# Exploring the diversity of blood-sucking Diptera in caves of Central Africa

**DOI:** 10.1038/s41598-017-00328-z

**Published:** 2017-03-21

**Authors:** Judicaël Obame-Nkoghe, Nil Rahola, Diego Ayala, Patrick Yangari, Davy Jiolle, Xavier Allene, Mathieu Bourgarel, Gael Darren Maganga, Nicolas Berthet, Eric-Maurice Leroy, Christophe Paupy

**Affiliations:** 1 Laboratoire MIVEGEC, UMR 224-5290 CNRS-IRD-UM, IRD Montpellier, France; 2 Centre International de Recherches Médicales de Franceville (CIRMF), Franceville, Gabon; 3 CIRAD, UPR AGIRs, F-34398 Montpellier, France; 4 Centre National de Recherche Scientifique (CNRS) UMR3569, Paris, France

## Abstract

Caves house pathogenic microorganisms, some of which are transmitted by blood-sucking arthropods. In Africa, previous studies identified mosquitoes, sand flies and biting midges as the main potential vectors of cave-dwelling pathogens. However, to understand their involvement in pathogen spillover, it is crucial to characterize their diversity, community composition and dynamics. Using CDC light traps, we collected hematophagous Diptera in six caves of Gabon during one-shot or longitudinal sampling, and investigated their species diversity and dynamics in relation with external rainfall. Overall, we identified 68 species of mosquitoes, sand flies and biting midges, including 45 new records for Gabon. The dominant species were: *Uranotaenia nigromaculata, Anopheles smithii s.l., Culex. rima* group and *Culex quasiguiarti* for mosquitoes, *Spelaeophlebotomus gigas* and *Spelaeomyia emilii* for sand flies and the *Culicoides trifasciellus* group and *Culicoides fulvithorax* for biting midges. The survey revealed that species assemblages were cave-specific and included mainly troglophilous and trogloxenous species. Both diversity and abundance varied according to the cave and sampling time, and were significantly associated with rainfall. These associations were modulated by the cave specific environmental conditions. Moreover, the presence of trogloxenous and troglophilous species could be of high significance for pathogen transfers between cave and epigeous hosts, including humans.

## Introduction

Cave ecosystems house many different pathogenic microorganisms, including opportunist pathogens, such as viruses, Haemosporidia, Trypanosomatida, bacteria and fungi^1–5^ that infect cave-dwelling vertebrates, especially bats^6–10^. Some of them require blood-sucking arthropod vectors for their transmission^11–14^. In Africa, the growing anthropization of caves (for mining, tourism, resource gathering or spiritual purposes) has increased the risk of spillover of emerging pathogens that naturally infect the fauna living inside the caves^6^. Studies carried out in African caves have helped inventorying the main potential vectors among mosquitoes (Culicidae), sand flies (Phlebotominae) and biting midges (Ceratopogonidae)^15–17^. Several cavernicolous mosquito species from the *Anopheles* (*An.*) and *Uranotaenia* (*Ur.*) genera, such as *An. vanhoufi* Wanson & Lebied*, An. rodhaini* Leleup & Lips*, An. faini* Leleup*, An. cavernicolus* Abonnenc*, An. vanthieli* Laarman*, An. caroni* Adam*, An. smithii, Ur. cavernicola* Mattingly, have been described^15^. Similarly, five sand flies species belonging to the *Spelaeophlebotomus* (*Sl.*) Theodor, *Phlebotomus* (*Ph.*) Loew and *Spelaeomyia* (*Sa*.) Theodor genera have been reported in Afrotropical caves, including *Sl. gigas* Parrot & Schewtz, *Sa. mirabilis* Parrot & Wanson*, Ph. balmicola* Abonnenc*, Sa. moucheti* Vattier-Bernard & Abonnenc and *Sa. emilii* Parrot and Wanson^18^. Conversely, little is known about cavernicolous biting midges. To date, only three species of the *Culicoides* genus (a genus that includes the predominant biting midges species known to be both hematophagous and potential vectors of pathogens^19^) have been reported in African caves: *C. grenieri* Vattier-Bernard & Adam, *C. rageaui* Vattier-Bernard & Adam and *C. brossetti* Vattier-Bernard & Adam^20, 21^. Although it has been suggested that these blood-sucking Diptera (mosquitoes, sand flies and biting midges) are potential important actors in the spillover of emerging diseases^22^, their role in pathogen epidemiology within cave ecosystems has rarely been investigated.

**Figure 1:**
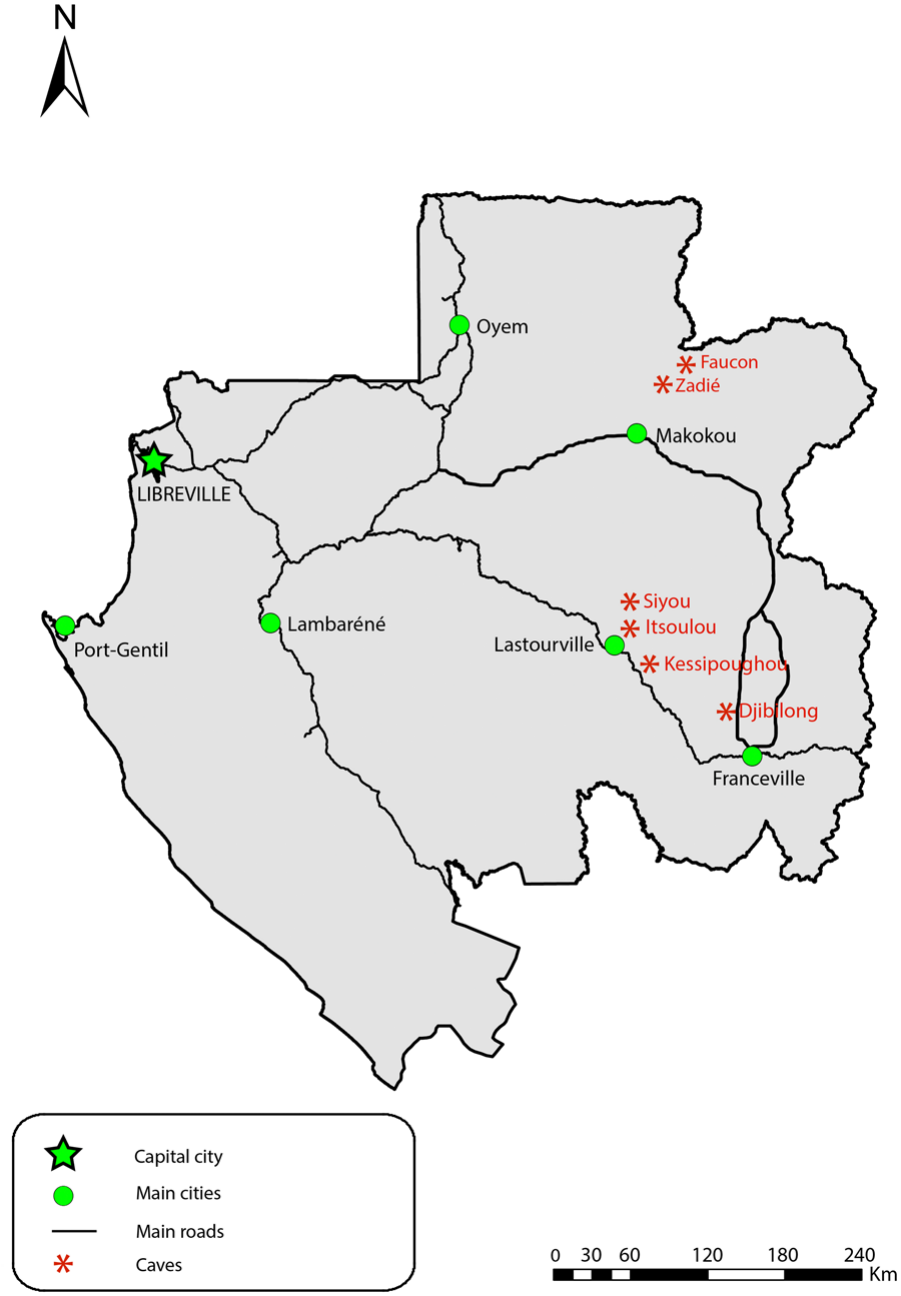
Geographic location of the six investigated caves. The map was created by co-authors using ArcGis 10.04.01 (https://desktop.arcgis.com/fr).

In Gabon, blood-sucking mosquitoes, sand flies and biting midges have previously been studied. With about 100 known species, mosquito taxa are the more documented^23^. Conversely, biting midges (six reported species: *C. austeni* Carter*, C. brossetti, C. brucei* Austen*, C. grahamii* Austen*, C. jouberti* Huttel, Huttel & Verdier*, C. tristanii* Huttel, Huttel & Verdier)^24^ and sand flies (five reported species: *Sl. gigas, Sergentomyia antennata* Newstead*, Se. schwetzi* Adler, Theodor & Parrot*, Se. africana* Newstead*, Ph. multihamatus* Rahola, Depaquit & Paupy)^25^ remain largely under-documented. In caves, blood-sucking dipterans have occasionally been studied, but only in the region of the Belinga mountains (North-East of the country). Therefore, except for the report of two mosquitoes (*An. smithii s.l.*, *Culex* sp. in Faucon cave), one sand fly (*Sl. gigas* in Zadie cave) and one biting midge (*C. brossetti* in Faucon cave) species^20, 26^, data on the diversity of cavernicolous insects of medical or veterinary interests are very limited. Moreover, almost nothing is known about their biology, population dynamics and community structure. To fill this gap, we performed an entomological survey in several caves of Gabon (Fig. 1). We focused on the diversity and population dynamics of Culicidae, Phlebotominae and Ceratopogonidae in relation with relevant environmental parameters, particularly external rainfall.

## Results

### Inventory and diversity of Diptera assemblages

During the study period, 4395 mosquitoes, 1449 sand flies and 363 biting midges were collected. In all sampled caves mosquitoes dominated the Diptera assemblages with the exception of Zadie cave where sand flies were predominant. The highest apparent density (*AD*) of mosquitoes (i.e., number of specimens per trap and per day) was observed in Faucon cave with a value of 39.5 (Table 1). Biting midges were observed only in Kessipoughou and Djibilong caves and showed low *AD* values (1.1 and 0.6 respectively) (Table 1).

**Table 1:** Density and diversity of the Diptera assemblages in the different caves.

	AD (sp/t/d)	S	Shannon index (H)	Estimated NS
**Culicidae**				
Kessipoughou	6.8	9	0.45	0.5
Djibilong	7.1	40	1.1	13.1
Faucon	39.5	3	0.1	0
Zadie	0.2	4	0.7	6
Siyou	4.2	8	0.8	1.5
Itsoulou	19.9	5	0.3	0
Total**	—	52	—	21.1
**Phlebotominae**				
Kessipoughou	1.3	6	0. 09	1.5
Djibilong	4.8	7	0.6	0
Faucon	0	0	—	0
Zadie	0.5	5	0.6	8
Siyou	3	1	0	0
Itsoulou	4.2	1	0	0
Total**	—	11	—	4.5
**Ceratopogonidae**				
Kessipoughou	1.1	2	0.01	0
Djibilong	0.6	5	0.6	0
Faucon	0	0	—	0
Zadie	0	0	—	0
Siyou	0	0	—	0
Itsoulou	0	0	—	0
Total**	—	5	—	0
**All Diptera groups**				
Kessipoughou	9.2	17	0.7	5
Djibilong	12.5	52	1.3	15.1
Faucon	39.5	3	0.1	0
Zadie	0.7	9	0.9	21
Siyou	7.2	9	0.8	0.5
Itsoulou	24.1	6	0.5	0
Total**	—	68	—	41.6

The number of sampling events was 44 in both Kessipoughou and Djibilong caves, 4 in Faucon, Zadie and Itsoulou caves, and 2 in Siyou cave. —, not applicable” or not calculated data; **“Total” refers to the total number of collected species for the species richness (S), and to the sum of non-sampled (NS) species for the NS column in all caves; AD, apparent density (number of specimens collected per trap and per day; sp/t/d); H, diversity index.

Mosquitoes belonged to 52 species (including 30 new records for Gabon) from 12 genera, particularly *Anopheles* (*An.*), *Culex* (*Cx.*) and *Uranotaenia* (*Ur.*) (Table 2). Eleven species of sand fly were observed belonging to four genera: *Spelaeophlebotomus* (*Sl.*), *Spelaeomyia* (*Sa.*), *Sergentomyia* (*Se.*) and *Phlebotomus* (*Ph.*). Eight of these sand fly species were never reported in Gabon before. Five biting midges species from the genera *Culicoides* (*C.*) and *Forcipomyia* (*F.*) were collected. All were new records for Gabon.

**Table 2:** Composition of mosquito, sand fly and biting midge species assemblages in the Gabonese caves under study.

Species	KESS	Number of specimens					Dominance index *d*					
		DJIB	FAUC	ZAD	SIY	ITSO	KESS	DJIB	FAUC	ZAD	SIY	ITSO
**Culicidae**												
*Ur. nigromaculata*	1114	126	62	0	11	28	0.7397	0.09	0.0654	0	**0.2156**	0.0877
Ur. cavernicola*^†^	189	0	1	1	8	12	**0.1254**	0	0.001	**0.25**	**0.1568**	0.0376
An. smithii s.l.**^†^	110	0	0	0	19	272	0.0730	0	0	0	**0.3625**	**0.8526**
*Cx. rima* group	65	244	886	0	6	6	0.0431	**0.155**	**0.9336**	0	**0.11**	0.019
An. faini*^†^	13	2	0	0	0	0	0.0086	0.001	0	0	0	0
Ur. nigripes*	11	0	0	0	0	0	0.0074	0	0	0	0	0
*An. funestus*	2	0	0	0	0	0	0.0014	0	0	0	0	0
*Cx. nebulosus*	1	0	0	0	0	0	0.0007	0	0	0	0	0
Cx. umbripes*	1	0	0	0	0	0	0.0007	0	0	0	0	0
Cx. quasiguiarti*	0	590	0	0	3	0	0	**0.376**	0	0	0.0571	0
Fi. uniformis*	0	136	0	0	0	0	0	0.086	0	0	0	0
Ur. caliginosa*	0	102	0	0	0	0	0	0.065	0	0	0	0
Ur. caeruleocephala*	0	76	0	0	0	0	0	0.048	0	0	0	0
Ur. machadoi*	0	75	0	0	0	0	0	0.047	0	0	0	0
Ur. bilineata*	0	65	0	0	0	0	0	0.041	0	0	0	0
*Cx. trifilatus*	0	56	0	0	0	0	0	0.035	0	0	0	0
*Lu. tigripes*	0	27	0	0	0	0	0	0.017	0	0	0	0
Cx. watti*	0	7	0	0	0	0	0	0.004	0	0	0	0
An. theileri*	0	7	0	0	0	0	0	0.004	0	0	0	0
*Ur. pallidocephala*	0	6	0	0	0	0	0	0.004	0	0	0	0
*An. marshallii*	0	4	0	0	0	0	0	0.002	0	0	0	0
*Ur. balfouri*	0	4	0	0	0	0	0	0.002	0	0	0	0
Ur. chorleyi*	0	3	0	0	0	0	0	0.002	0	0	0	0
Co. pseudoconopas*	0	3	0	0	0	0	0	0.002	0	0	0	0
Cx. andersoni*	0	3	0	0	0	0	0	0.002	0	0	0	0
*Mi. plumosa*	0	3	0	0	0	0	0	0.002	0	0	0	0
*Co. aurites*	0	2	0	0	0	0	0	0.001	0	0	0	0
*Culex sp.*	0	2	0	1	0	0	0	0.001	0	**0.25**	0	0
An. jebudensis*	0	2	0	0	0	0	0	0.001	0	0	0	0
*An. obscurus*	0	2	0	0	0	0	0	0.001	0	0	0	0
Cx. zombaensis*	0	2	0	0	0	0	0	0.001	0	0	0	0
Er. grahami*	0	2	0	0	0	0	0	0.001	0	0	0	0
*Ca. argenteopunctata*	0	2	0	0	1	0	0	0.001	0	0	0.0196	0
Cx. cinerellus*	0	1	0	0	0	0	0	0.0006	0	0	0	0
Finlaya sp.*	0	1	0	0	0	0	0	0.0006	0	0	0	0
*An. nili*	0	1	0	0	0	0	0	0.0006	0	0	0	0
Co. versicolor*	0	1	0	0	0	0	0	0.0006	0	0	0	0
Cx. semibrunneus*	0	1	0	0	0	0	0	0.0006	0	0	0	0
*Cx. annulioris*	0	1	0	0	0	0	0	0.0006	0	0	0	0
Co. microannulata*	0	1	0	0	0	0	0	0.0006	0	0	0	0
An. natalensis*	0	1	0	0	0	0	0	0.0006	0	0	0	0
Cx. simpsoni*	0	1	0	0	0	0	0	0.0006	0	0	0	0
*Er. chrysogaster*	0	1	0	0	0	0	0	0.0006	0	0	0	0
An. schwetzi*	0	1	0	0	0	0	0	0.0006	0	0	0	0
Ur. alboabdominalis*	0	1	0	0	0	0	0	0.0006	0	0	0	0
Ps. kummi*	0	1	0	0	0	0	0	0.0006	0	0	0	0
Cx. cinereus*	0	1	0	0	0	0	0	0.0006	0	0	0	0
*Aedes sp.*	0	0	0	1	0	0	0	0	0	**0.25**	0	0
*Anopheles sp.*	0	0	0	1	0	0	0	0	0	**0.25**	0	0
*Cx. rubinotus*	0	0	0	0	2	0	0	0	0	0	0.0392	0
*Ur. mashonaensis*	0	0	0	0	1	0	0	0	0	0	0.0196	0
Ae. simulans*	0	0	0	0	0	1	0	0	0	0	0.0196	0.0031
**Phlebotominae**												
Sl. gigas**^†^	268	428	0	6	37	68	**0.9675**	**0.4056**	0	**0.5**	**1**	**1**
*Sa. emilii* ^†^	4	352	0	1	0	0	0.0145	**0.3336**	0	0.0834	0	0
*Se. bedfordi* group*	2	12	0	0	0	0	0.0072	0.0114	0	0	0	0
Ph. rodhaini*	1	214	0	0	0	0	0.0036	**0.2029**	0	0	0	0
Se. ingrami*	1	46	0	0	0	0	0.0036	0.0437	0	0	0	0
Se. dubia*	1	0	0	0	0	0	0.0036	0	0	0	0	0
Se. congolensis*	0	0	0	3	0	0	0	0	0	**0.25**	0	0
Se. africana*	0	0	0	1	0	0	0	0	0	0.0833	0	0
Se. magna*	0	0	0	1	0	0	0	0	0	0.0833	0	0
*Sa. moucheti* ^†^	0	1	0	0	0	0	0	0.0009	0	0	0	0
Se. balmicola*	0	2	0	0	0	0	0	0.0019	0	0	0	0
**Ceratopogonidae**												
*C. trifasciellus* group*	240	17	0	0	0	0	**0.9917**	**0.1405**	0	0	0	0
Forcipomyia sp*	2	30	0	0	0	0	0.0083	**0.2479**	0	0	0	0
C. fulvithorax*	0	65	0	0	0	0	0	**0.5372**	0	0	0	0
*C. milnei* group*	0	8	0	0	0	0	0	0.0662	0	0	0	0
C. distinctipennis*	0	1	0	0	0	0	0	0.0082	0	0	0	0

**KESS**: Kessipoughou, **DJIB**: Djibilong, **FAUC**: Faucon, **ZAD**: Zadie, **SIY**: Siyou, **ITSO**: Itsoulou. ***Ur:*** *Uranotaenia*, ***An:*** *Anopheles*, ***Cx:*** *Culex*, ***Fi:*** *Ficalbia*, ***Lu:*** *Lutzia*, ***Co:*** *Coquillettidia*, ***Mi:*** *Mimomyia*, ***Er:*** *Eretmapodites.* ***Ca:*** *Catageiomyia*, ***Ps:*** *Pseudoarmigeres*, ***Ae:*** *Aedes.* ***Sl:*** *Spelaeophlebotomus*, ***Sa:*** *Spelaeomyia*, ***Se:*** *Sergentomyia*, ***Ph:*** *Phlebotomus*, ***C:*** *Culicoides.* *New records for Gabon; **species previously recorded in Gabonese caves; ^†^Species previously known as “true cavernicolous”. The index *d* value of dominant species is marked in bold.

The number of species collected in the different caves (i.e., species richness *S*) ranged from 3 (Faucon) to 40 (Djibilong) for mosquitoes, and from 0 (Faucon) to 7 (Djibilong) for sand flies. For biting midges, only 2 and 5 species were collected in Kessipoughou and Djibilong, respectively, during the entire study (Table 1). Mosquitoes appeared to be more diversified in the caves of Djibilong (*S* = 40, and Shannon diversity index *H* = 1.1) and Siyou (*S* = 9, *H* = 0.8) than in the other caves. The diversity of sand flies was highest in Djibilong (*S* = 7, *H* = 0.6) and Zadie (*S* = 5, *H* = 0.6) and of biting midges in Djibilong cave (*S* = 5, *H* = 0.6).

**Figure 2:**
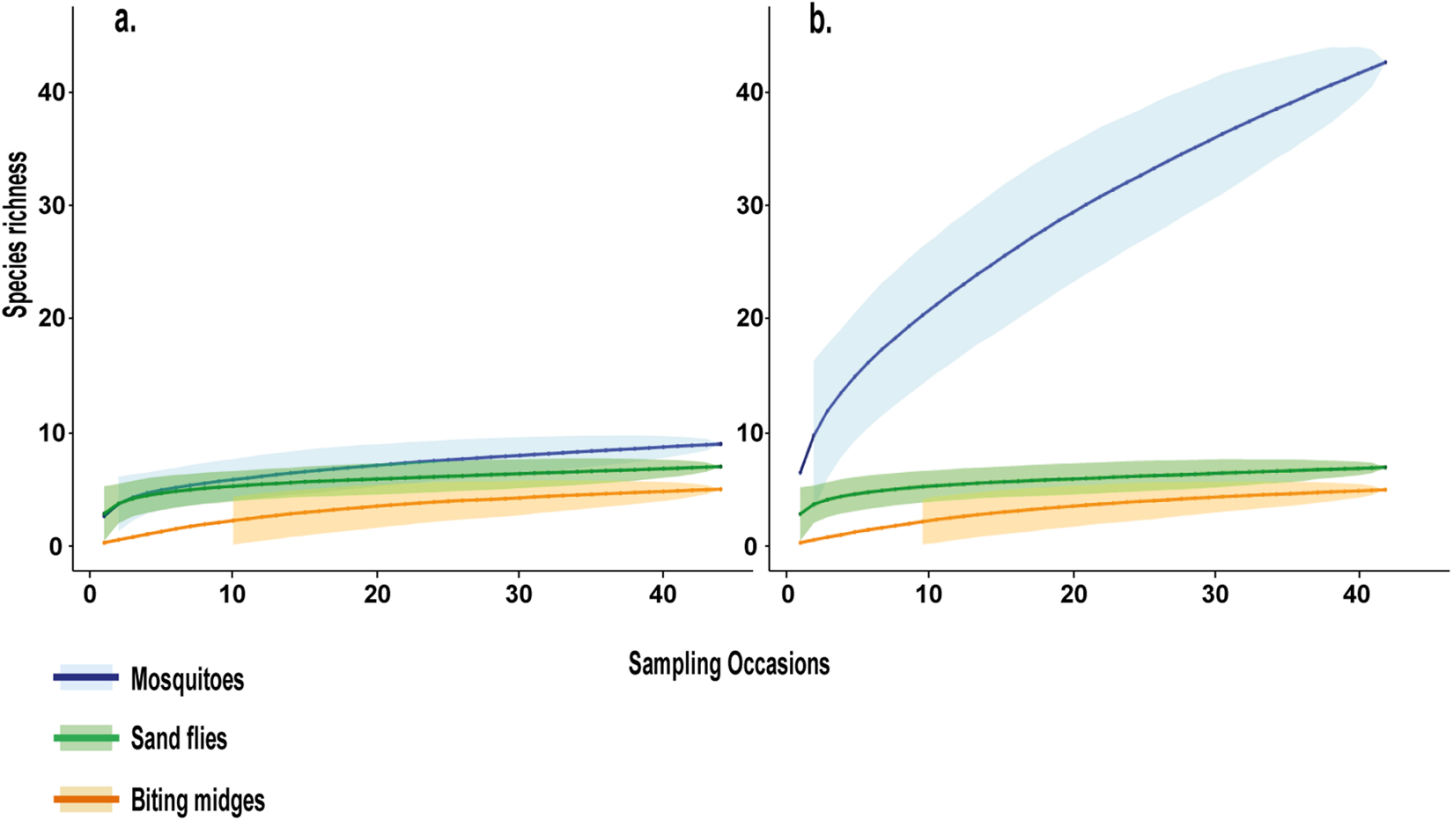
Species accumulation curves for mosquitoes, sand flies and biting midges relative to the number of sampling events in Kessipoughou (**a**) and Djibilong (**b**) caves. Curves show the cumulated species richness (S values) of Diptera groups according to sampling events. Sampling representativeness seemed good for each insect group in both caves, with the exception of mosquitoes in Djibilong. The lack of representativeness for the mosquito community inside Djibilong cave was confirmed by the important number of estimated non-sampled species (*NS* = 13.1, Table 1), extrapolated from the Chao index.

To assess whether sampling was representative of the species assemblages living in Kessipoughou and Djibilong caves, species accumulation curves (as an estimate of species richness) were plotted for 44 sampling events (Fig. 2). Except for mosquitoes in Djibilong cave, sampling representativeness seemed good for each insect group in both caves. The lack of representativeness for the mosquito community inside Djibilong cave was confirmed by the important number of estimated non-sampled species (*NS* = 13.1, Table 1), extrapolated from the Chao index (a species richness estimator)^27^. The *NS* value was particularly valuable for the caves were insects were collected by one-shot sampling. Indeed, in Zadie cave it was 6.0 for mosquitoes and 8.0 for sand flies, suggesting that a significant number of species escaped capture with our sampling procedure. Conversely, it was zero or close to zero for the Faucon, Itsoulou and Siyou caves where insects were also captured by one-shot sampling (Table 1).

Concerning each Diptera group, species assemblages greatly differed between caves for mosquitoes and biting midges (Morisita-Horn similarity index *C* mostly < 0.5) (Supplementary Table S1), but not for sand flies (*C* > 0.6) (Supplementary Table S1). For each Diptera group, we assumed that species assemblages differed between caves where Diptera were observed and caves where they were not, despite the NA values (Supplementary Table S1). The species composition of communities and also the dominance pattern considerably varied among caves (Table 2). Among mosquitoes, *An. smithii s.l.* was the most dominant species in Siyou (*d* = *0.37*) and Itsoulou (*d* = *0.85*), *Ur. nigromaculata* in Kessipoughou (*d* = *0.73*), *Cx. quasiguiarti* in Djibilong (*d* = *0.37*) and *Cx. rima* group in Faucon (*d* = *0.93*). In all caves, *Sl. gigas* was the dominant sand fly species (*d* ranged from 0.4 to 1), followed by *Sa. emilii* (*d* ranged from 0 to 0.3), with the exception of Zadie cave where *Se. congolensis* was the second dominant species (*d* = *0.25*). Biting midges were represented mainly by the *C. trifasciellus* group in Kessipoughou (*d* = *0.99*) and by *C. fulvithorax* in Djibilong cave (*d* = *0.53*).

Among all collected mosquitoes, only two species (3.8%; *Ur. cavernicola* and *An. faini*) were previously known to be restricted to caves ecosystems and were defined as “true cavernicolous” (i.e., troglobitic species that spend their entire life cycle exclusively in caves) (Table 2). The proportion of true cavernicolous species was higher among the collected sand flies (4/11; 36.3%; *Sl. gigas, Sa. emilii, Sa. moucheti* and *Se. balmicola*) (Table 2). None of the collected biting midges was previously found in caves.

### Spatio-temporal dynamics of Diptera assemblages

**Figure 3:**
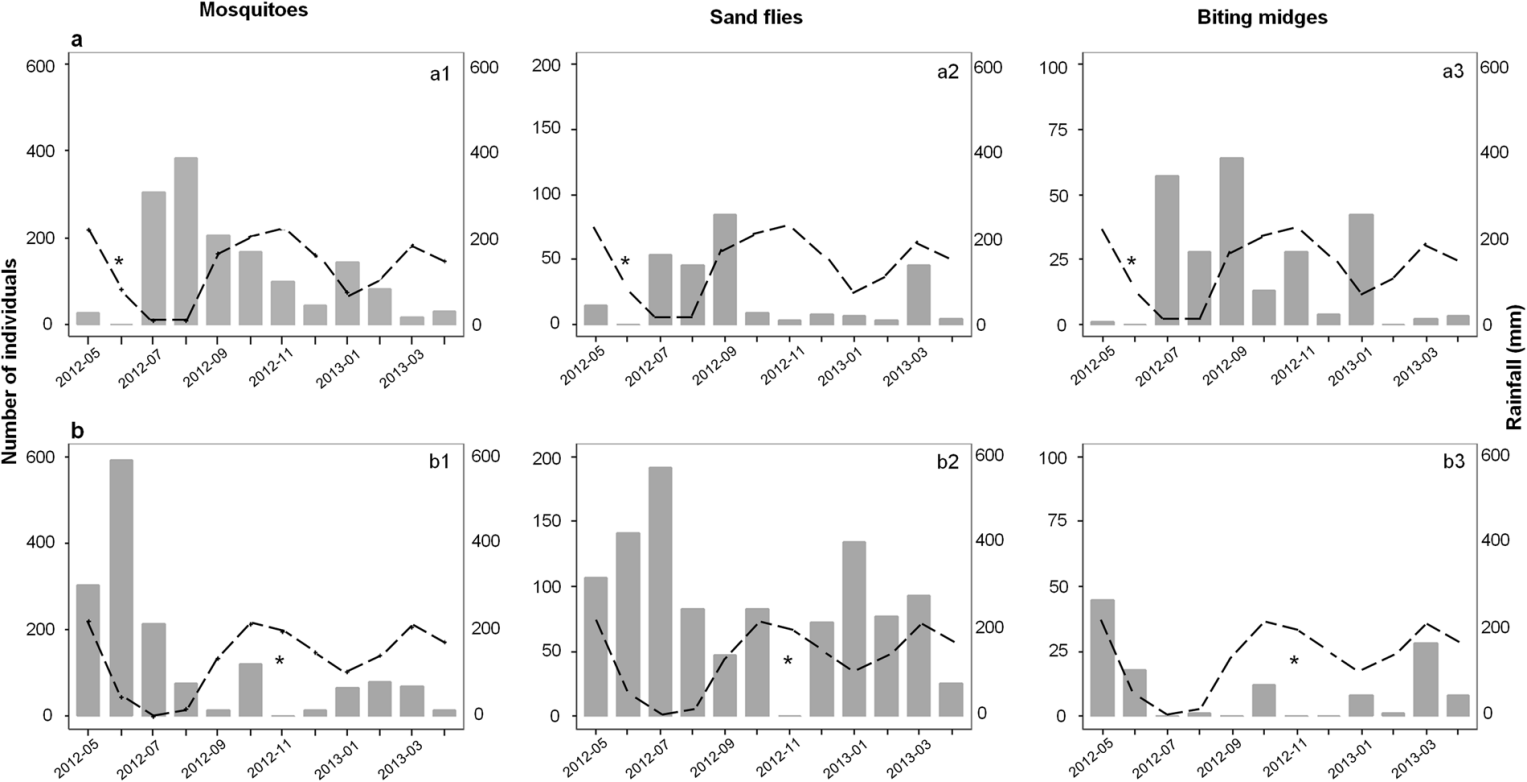
Density variations for each Diptera group along time in Kessipoughou (**a**) and Djibilong (**b**) caves. Histograms (in grey) show the number of individuals for each Diptera group (mosquitoes, sand flies and biting midges) collected each month from May 2012 to April 2013. The monthly rainfall estimates (in mm, broken line) were from the United States Department of Commerce, National Weather Service/Climate Prediction Center (http://www.cpc.ncep.noaa.gov/products/fews/africa). *Month without sampling.

**Figure 4:**
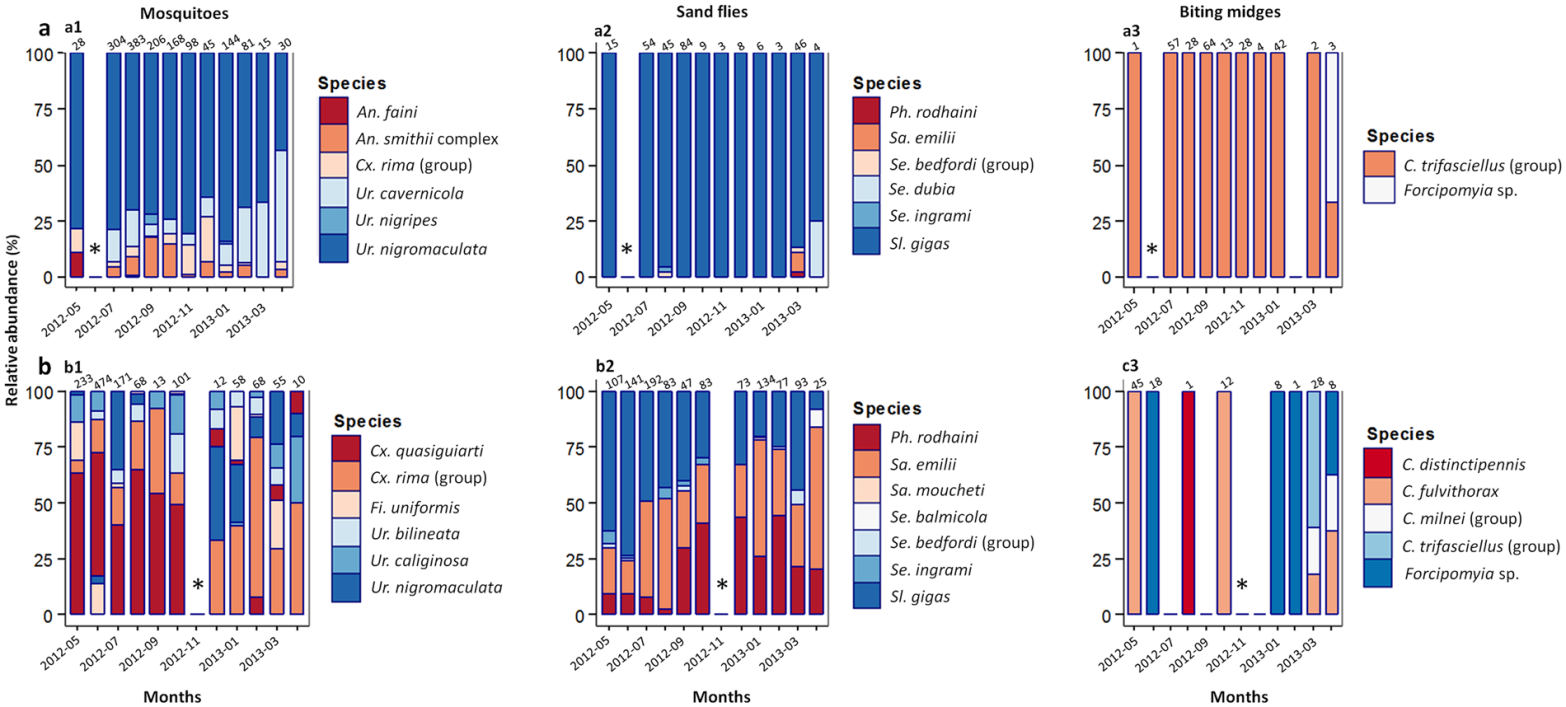
Temporal variations in species dominance in Kessipoughou (**a**) and Djibilong (**b**) caves. For mosquitoes, only the six most dominant species in assemblages were taken into account (most of the other species represented less than 0.2% in density/each). For all Diptera, species with the highest relative abundance at a given time point were considered as dominant. The number of individuals collected each month is shown above the bar. *Month without sampling. gr., group.

Comparison of the *H* values for the three Diptera groups collected by longitudinal sampling showed that diversity was significantly higher in Djibilong than in Kessipoughou cave (Supplementary Fig. S1). Analysis of the community dynamics of each Diptera group in both caves revealed major density variations along time (Fig. 3). Although mosquitoes were sampled in both caves throughout the year, the highest densities were observed between July and October in Kessipoughou (Fig. 3a1), and between May and July in Djibilong cave (Fig. 3b1). Moreover, the species composition of mosquito assemblages in both caves varied over time and some drastic shifts in species dominance were observed (Fig. 4). In Kessipoughou cave, *Ur. nigromaculata* was the predominant species from May 2012 to March 2013 and then was overtaken by *Ur. cavernicola* in April 2013 (Fig. 4a1). In Djibilong cave, *Cx. quasiguiarti* was predominant from May 2012 to October 2012, *Ur. nigromaculata* in December 2012, and the *Cx. rima* group from January 2012 to April 2013 (Fig. 4b1). Conversely, the diversity of mosquito species (mean *H* value) varied significantly over time only in Djibilong cave (Supplementary Fig. S2). Rainfall was negatively associated with mosquito diversity in Djibilong (*t-value* = −3.3, *p* = 0.0008) and with mosquito density in Kessipoughou cave (*t-value* = −3.6, *p* = 0.0004).

Sand flies also were collected in both caves throughout the year; however, their density was higher from July to September and in March in Kessipoughou cave (Fig. 3a2), and from June to July and in January in Djibilong cave (Fig. 3b2). *Sl. gigas* was the predominant species during the entire survey period in Kessipoughou cave (Fig. 4a2). Conversely, a more complex dominance pattern with dominance shifts from *Sl. gigas to Ph. rodhaini* and *Sa. emilii* was observed in Djibilong cave (Fig. 4b2). Sand fly diversity (mean *H* value) did not significantly change over time in both caves (Supplementary Fig. S2). In Djibilong cave, rainfall was negatively associated with sand fly density (*t-value* = −2.9, *p* = 0.003).

The density of biting midges also varied over time in both caves with peaks of abundance in July, September and January in Kessipoughou (Fig. 3a3), and in May and March in Djibilong cave (Fig. 3b3). In Kessipoughou cave, members of the *C. trifasciellus* group were predominant all year around, except in April when they were overtaken by *Forcipomyia* spp. (Fig. 4a3). In Djibilong cave, no significant species predominance was detected during the entire survey (Fig. 4b3). The mean *H* value of biting midges did not significantly change over time in both caves (Supplementary Fig. S2) and no significant association between biting midge density/diversity and rainfall was observed.

## Discussion

In all the caves we investigated in Gabon, Diptera assemblages were always dominated by mosquitoes, except in Zadie cave where sand flies were the most abundant. This observation suggests that these caves offer conditions that are suitable particularly for mosquito development. On the other hand, the micro-environmental conditions within Zadie cave could be less suitable for mosquitoes or biting midges than for sand flies, as previously observed^26^. Indeed, Zadie cave is relatively drier than the other explored caves, with few potential breeding sites for mosquitoes or biting midges (their immature stages need a humid substratum), whereas sand flies could easily breed on the powdery substratum, despite the lower relative humidity^26^. Moreover, in all explored caves, we collected Ceratopogonidae. However, we recorded blood-feeding species (i.e., belonging to the genera *Culicoides* and *Forcipomyia*) only in Kessipoughou and Djibilong caves. Although a previous study reported *Culicoides brossetti* species that belong to the *Culicoides trifasciellus* group in the deepest zone of Faucon cave^20^, we did not capture *C. brossetti* or C. *brossetti*-like individuals in Faucon cave (where we conducted one-shot sampling in January and February year). It could be possible that this sampling time was not suitable for capturing individuals of the *C. trifasciellus* group (i.e., *C. brossetti* or C. *brossetti*-like) in Faucon cave, thus explaining their absence in our study. However, even in Kessipoughou and Djibilong caves where we carried out longitudinal sampling, the density of blood-feeding Ceratopogonidae was lower than that of mosquitoes and sand flies. Therefore, blood-feeding Ceratopogonidae (e.g., *C. trifasciellus* group) could be present also in the other caves, but at very low density. Longitudinal sampling in these caves could bring more insights into this issue.

Our study allowed us to significantly increase the number of recorded species in Gabon. Specifically, our work adds 50 Culicidae species (including 30 new records for Gabon) to the two previously known mosquito species collected in Gabonese caves. *Ur. nigromaculata* and the *Cx. rima* group dominated the mosquito species assemblages. Similarly, we found 11 species of sand flies (including 8 new records for Gabon) in these caves, compared with the only five species previously known in Gabon^25^, but *Sl. gigas* was the dominant one. Finally, the community of cavernicolous biting midges included five taxa, all new for the country and dominated by the *C. trifasciellus* group.

Some of the species reported here have been previously found in caves of other African countries. For instance, the mosquito species *Ur. cavernicola, An. smithii s.l.* and *An. faini* have been previously collected in caves of Democratic Republic of Congo and Cameroon^12, 15, 28, 29^. Similarly, among the identified sand fly species, *Sl. gigas* is widely distributed throughout Africa, whereas *Sa. emilii* have been previously recorded in Congo-Brazzaville, Cameroon and Gabon, *Se. balmicola* in Congo-Brazzaville, Cameroon and Gabon, and *Sa. moucheti* in Cameroon, Central African Republic and Gabon^17, 30^. Conversely, the two biting midge species we detected (*C. grenieri* and *C. rageaui*) have never been reported in African caves before^21^. Our analysis (particularly, species accumulation curves and NS values) suggests that sampling was exhaustive for all Diptera groups and in all caves, except for mosquitoes in Zadie cave, probably due to the low number of captured individuals, and in Djibilong cave where the extrapolated number of non-sampled species was quite important. Thus, with more than 50% of all known mosquito species in Gabon (in any ecosystems) and several new records of sand flies and biting midges, this study improves the knowledge about cavernicolous blood-sucking Diptera in Gabon and in Central Africa.

We observed that Kessipoughou cave is a very deep cave, less opened to the outside. In this cave, there are several mosquito breeding sites and large colonies of bats (a potential major blood source) that might favor the development of only few potentially well adapted troglophilous or troglobitic species. This could explain the high density and low diversity observed in this cave. Conversely, Djibilong cave is less deep and more opened to the outside, thus favoring the entry of several species from the outside environment and explaining the high diversity observed. Therefore, the difference in mosquito assemblages observed in these two caves could be the consequence of these habitat differences.

In this study, potential true cavernicolous (troglobitic) species were generally a minority compared with the other species. Indeed, the proportion of previously known true cavernicolous species was very low (3.8%) for mosquitoes and low (36.3%) for sand flies. *Anopheles smithii s.l.* was known to be restricted to caves, although it has been occasionally recorded inside human habitations in Koulamoutou, Gabon^31^. Some species from epigeous environments showed high densities inside the six caves under study (*Ur*. *nigromaculata*, *Cx*. *rima* group and *Cx*. *quasiguiarti* among mosquitoes; *Ph*. *rodhaini* and *Se*. *congolensis* among sand flies; *C*. *trifasciellus* group among biting midges), suggesting that they breed in this environment. Therefore, these opportunistic species could be considered as troglophilous (i.e., capable of breeding in epigeous and also in cavernicolous environments). More accurate investigations, including larval surveys and feeding behavior studies, should allow a better determination of their cavernicolous status. Besides these species, all the others, including biting midges, have been previously recorded only in epigeous habitats^32–42^, suggesting that they are trogloxenous (i.e., living and breeding outside caves) and use cave temporarily to rest (adults) or to seek cavernicolous hosts. The presence inside caves of many trogloxenous and troglophilous species that could move between the inside of the caves and the outside environment might favor the externalization of pathogens that infect cave-dwelling hosts (e.g., bats) and their transfer, through bridge vectors, to animals or humans living in the surroundings of caves, or animals living in different caves (e.g., populations of bat using different caves). Indeed, the Siyou and Itsoulou caves are very close to Lastoursville, and Djibilong cave is located within a ranch. Therefore, they represent favorable contexts for the spillover of cave-dwelling Diptera-borne zoonotic pathogens to humans or livestock through bridge vectors.

Our results show that sand fly assemblages were mainly composed of troglophilous or troglobitic species^43^ and were similar between caves, except for a couple of caves, particularly Faucon cave where no sand fly was recovered. It suggests that the internal micro-environmental conditions (e.g., cave physical nature and breeding site types and densities) required for the development of cavernicolous sand flies might be comparable in all the studied caves, except for the Faucon cave that presents very different micro-climatic conditions compared with the other caves^44^. Conversely, the assemblages of mosquito and biting midge species were cave-specific, suggesting that the micro-environmental conditions required for their presence and/or their development may differ among caves. As these assemblages include trogloxenous and troglophilous species, variations in the external environmental conditions might affect the nature of the Diptera community around each cave and consequently, also the composition of the communities within each cave.

Moreover, longitudinal sampling revealed significant quantitative and qualitative fluctuations in both mosquito and sand fly assemblages over time. This generates shifts of species dominance that could be explained by micro-environmental changes within (e.g., nature and availability of larval breeding sites for true cavernicolous species) and in the surroundings of the caves (trogloxenous species).

In Kessipoughou cave, rainfall was negatively associated with mosquito density. In this cave, dominated by true cavernicolous species, mosquito larvae mainly breed in small water rock-pools along the riverbanks (personal observation). During periods of heavy rainfall, the stream level rises and rock-pools containing immature stages are flooded, leading to a decrease of the mosquito population density, as documented in epigeous environments^45–47^. In Djibilong cave, where trogloxenous species represent an important part of the mosquito assemblages, rainfall negatively influenced mosquito diversity rather than density. This could be explained by the fact that external species readily enter during dry periods, probably guided by physiological needs (e.g., for aestivation).

Rainfall also negatively affected sand fly density in Djibilong. For breeding, sand flies need a wet substratum (i.e., moist soil); however in this cave, during the rainy season nearly all the floor surface is flooded (personal observation), thus limiting the number of suitable breeding places and leading to a drop of the population size.

Our analysis also highlights that some Diptera species reach sufficient densities to support the transmission of pathogens insides caves. Only few of them have been previously shown to transmit pathogens in caves. For instance, *An. smithii s.l.* ensures the transmission of Plasmodiidae parasites to cave-dwelling vertebrates^48^. Mosquitoes could also serve as vectors for arboviruses because various species of both genera are known vectors of arboviruses in Africa, such as Rift Valley fever virus, West Nile virus and others^49, 50^. Particular attention should be paid to sand flies because in Africa, this group includes vectors of *Leishmania* parasites (particularly the genus *Phlebotomus* ^51^) and of viruses of the Bunyaviridae, Rhabdoviridae and Flaviviridae families^52^. Biting midges, which are known vectors of animal pathogens, such as haemosporidian parasites of *Hepatocystis* and *Nycteria* genera and arboviruses of the Reoviridae or Rhabdoviridae families^53, 54^, could also serve as vectors in caves. Therefore, it would be interesting to develop research programs to assess the presence of pathogens in cave-dwelling Diptera and to precisely evaluate the medical or veterinary risk related to the anthropization of caves and their surroundings. The evaluation of such risk requires also studying the blood feeding patterns of cavernicolous Diptera, particularly in order to determine whether some species could bite external vertebrate hosts (including humans) within or outside the cave, thus transferring cave-dwelling pathogens. As our study indicates that a significant proportion of Diptera found inside caves are trogloxenous and troglophilous, it is now important to assess whether and how these species can bridge pathogens from cavernicolous reservoirs to humans or domestic animals, especially for caves located in inhabited areas, such as Siyou, Itsoulou and Djibilong. Indeed, several of the collected species (at least twenty mosquito species, including *An. funestus, An. marshallii, An. nili, Cx. nebulosus, Cx. simpsoni, Fi. uniformis, Ur. bilineata, Ur. caeruleocephala* and *Ur. Mashonaensis*; five sand fly species: *Sl. gigas, Ph. rodhaini, Se. bedfordi* group, *Se ingrami* and *Se. magna*; and all the *Culicoides* species recorded in this study) can feed on a wide range of mammals, including wild or domestic animals and humans^18, 40, 55–65^.

## Methods

### Study areas

Mosquitoes, sand flies and biting midges were collected inside six caves that are located in the eastern part of Gabon (Fig. 1) and are among the most anthropized in this country, mainly for mining, hunting and tourism purposes. The Faucon (01.07287N 13.20739 E) and Zadie (00.98595N 13.19745 E) caves are in the heart of the Belinga Mountains, whereas the Kessipoughou cave (00.86722S 12.77389 E), which is currently considered one of the biggest known caves in Gabon, is in a forested area in the middle east of the country, near Lastoursville. The Siyou (00.80889S 12.76334 E) and Itsoulou (00.80639S 12.77389 E) caves also are in the rainforest around Lastoursville. The Djibilong cave (01.36261 S13.46296 E) is located in a patch of forest surrounded by savanna, north of Franceville. More details about these caves were previously published^44^. All studied caves are characterized by the presence of bat colonies and all of them, except Zadie cave, are crossed by internal free-flowing rivers (Kessipoughou, Itsoulou, Siyou) or contain stagnant water ponds of variable size and depth, depending on the season (Djibilong and Faucon). In Zadie cave, the environment is drier because water ingress or seepage is very limited during the rainy seasons.

### Insect sampling and species identification

Arthropods were collected using CDC light traps without CO_2_ during one-shot sampling in the Faucon, Zadie, Siyou and Itsoulou caves and longitudinal sampling in the Kessipoughou and Djibilong caves. Four (Faucon, Zadie, Siyou and Itsoulou) to five traps (Kessipoughou and Djibilong) were positioned in each cave, taking care to minimize the competition between them. In Kessipoughou and Djibilong, trap positions remained fixed throughout the duration of the longitudinal survey. Traps were turned on during: 1) 48 consecutive hours per month in Faucon, Zadie (January 2011 to February 2011) and Siyou (August 2013); 2) 96 consecutive hours per month in Itsoulou (August 2013) as well as in Kessipoughou and Djibilong (11 months between May 2012 and April 2013). Overall, the total trapping effort was of 11,904 hours. Collection bags were replaced each 24 hours and placed at −20 °C for 1 hour to kill the collected insects that were subsequently sorted in mosquitoes, sand flies and biting midges. Mosquitoes were morphologically identified (species or group of species) using “homemade” taxonomic keys based on updates of the Edwards’ identification keys for Ethiopian mosquitoes^32^. Species were named according to the on-line list of valid species (http://mosquito-taxonomic-inventory.info). Sand flies and biting midges were morphologically identified by observation of head, wings, genitalia and spermatheca using a microscope. The body parts used for identification were dissected and ephemerally mounted in Marc-André solution^66^ heated at 60 °C. The taxonomic identification of sand flies and biting midges was done using the keys for African Phlebotominae^66^ and African Ceratopogonidae, respectively^19, 37, 40^.

### Data analysis

All statistical analyses were performed using *R* v*3.0.2* (https://www.r-project.org/). To determine the sampling efficiency in the Kessipoughou and Djibilong caves, species accumulation curves were plotted according to a randomization procedure using the *vegan* package^67^ and by fixing the number of permutations to 1000. The apparent density (*AD*) of insects was estimated for all insect groups as the number of specimens collected per trap and per day (sp/t/d). Species richness (*S*) was determined as the number of insect species collected. In addition to (*S*), the diversity of communities was assessed using the Shannon index (*H*)^68^ calculated with the “*diversity*” command of the *vegan* package. For each cave, the number of non-sampled species (*NS*) was extrapolated by estimating the Chao index^27^ using the “*estimateR*” command of the *vegan* package. The dominant species index (*d*) in each group was estimated using the Berger-Parker equation^69^: *d* = *N*_*i*_ */N*, where *N*_*i*_ is the number of individuals of the ${i}$ ^th^ species and *N* the total number of sampled individuals (all species). It ranges from 0 to 1, and *d* values close to 1 indicate high dominance.

To investigate the cave similarity in terms of species composition and density, the Morisita-Horn similarity index (*C*)^70^ between sites was calculated using the “*vegdist*” command of the *vegan* package. Because “*vegdist*” is an analysis of dissimilarity (*C’*), *C* = *1* − *C’* was used for this study. *C* ranges from 0 (0% of similarity) to 1 (100% of identity between sites).

The relationships between monthly rainfall (chosen as environmental variable) and the variations of insect density and diversity indices during the study period were analysed in the Kessipoughou and Djibilong caves. To this aim, Generalized Linear Models (GLM) were fitted with identity links for each insect group using the *lme4* package^71^ and monthly “*rainfall*” was used as explanatory variable. The monthly estimates of accumulated precipitations were from the United States Department of Commerce, National Weather Service/Climate Prediction Center (http://www.cpc.ncep.noaa.gov/products/fews/africa). Data for each cave were retrieved using their GPS coordinates.

## Electronic supplementary material

Supplemental File 1
