# Supplementary material for: Exploring the diversity of blood-sucking Diptera in caves of Central Africa
Source: Sci Rep. 2017 Mar 21;7:250. doi: 10.1038/s41598-017-00328-z (PMC5428272; doi:10.1038/s41598-017-00328-z)

**Exploring the diversity of blood-sucking Diptera in caves of Central Africa**

Judicaël Obame-Nkoghe, Nil Rahola, Diego Ayala, Patrick Yangari, Davy Jiolle, Xavier Allene, Mathieu Bourgarel, Gael Darren Maganga, Nicolas Berthet, Eric-Maurice Leroy and Christophe Paupy

**Table S1**: **Comparison of the similarity index *C* values for each Diptera group between caves**

| **Culicidae** | | | | | |  | **Phlebotominae** | | | | | |
| --- | --- | --- | --- | --- | --- | --- | --- | --- | --- | --- | --- | --- |
|  | Djibilong | Kessipoughou | Faucon | Zadie | Siyou |  |  | Djibilong | Kessipoughou | Faucon | Zadie | Siyou |
| Kessipoughou | 0.17 |  |  |  |  |  | Kessipoughou | 0.63 |  |  |  |  |
| Faucon | 0.28 | 0.12 |  |  |  |  | Faucon | Na | Na |  |  |  |
| Zadie | <0.01 | 0.11 | <0.01 |  |  |  | Zadie | 0.78 | 0.80 | Na |  |  |
| Siyou | 0.28 | 0.53 | 0.22 | 0.46 |  |  | Siyou | 0.61 | 0.99 | Na | 0.78 |  |
| Itsolu | 0.02 | 0.20 | 0.03 | 0.02 | 0.72 |  | Itsolu | 0.61 | 0.99 | Na | 0.78 | 1 |

**Na**, couples in which mosquitoes or sand flies were not observed in at least one of the two caves.

Biting midges were only collected in Kessipoughou and Djibilong and the similarity index was 0.16.

**Fig. S1. Comparison of the mean Shannon diversity index *H* for Culicidae (mosquitoes), Phlebotominae (sandflies) and Ceratopogonidae (biting midges) in Kessipoughou and Djibilong caves.** The Shapiro-Wilk test of normality (*alpha* level of 0.05) showed that the data for 11 months of observation were non-normally distributed (all p-value were <0.05). Therefore, the mean diversity index *H* values for the three groups for the entire sampling period were then compared between caves using the non-parametric alternative Wilcoxon test with an *alpha* level of 0.05. "W" indicates the Wilcoxon comparison test value.


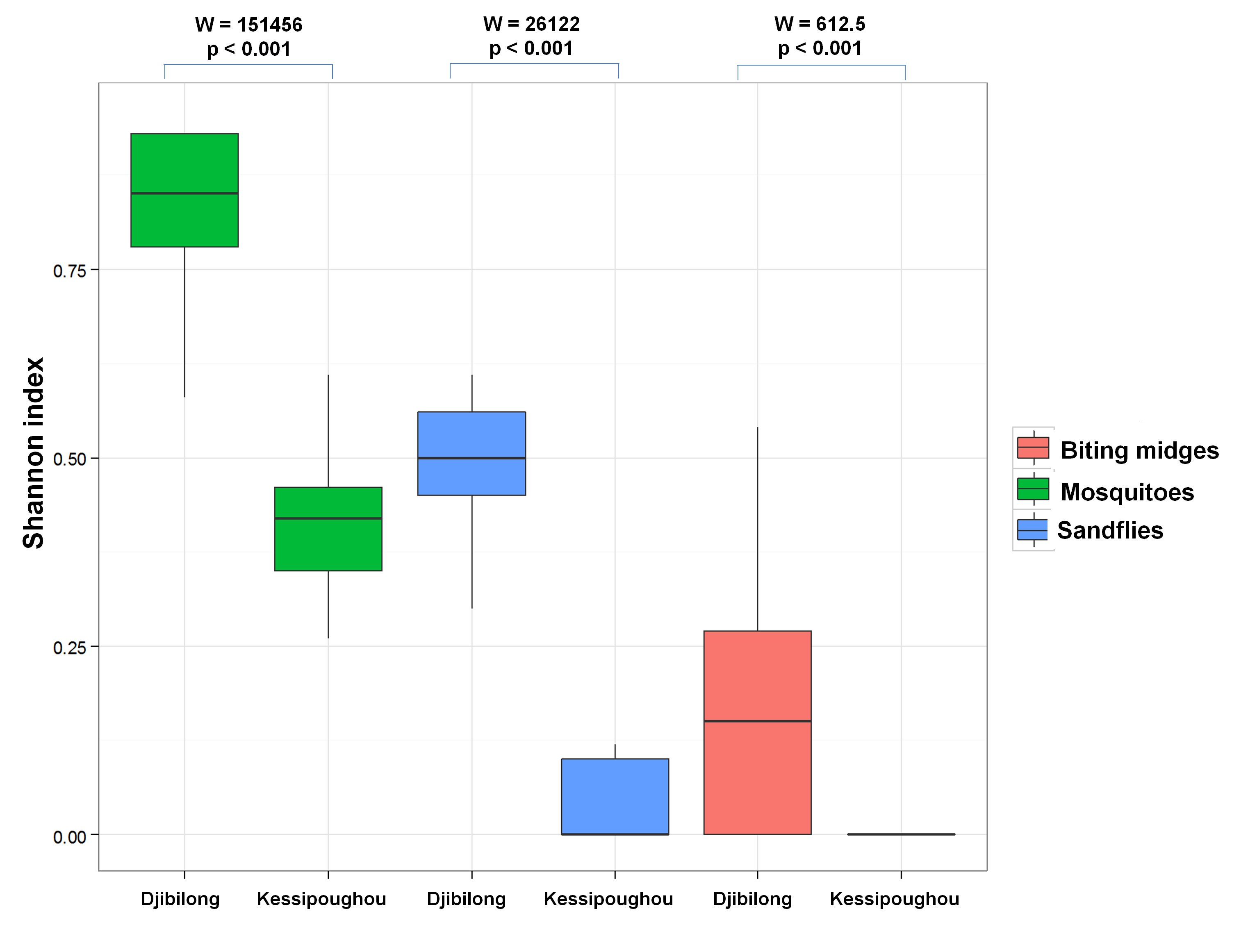


**Fig. S2. Variations of the mean values of the Shannon diversity index *H* for mosquitoes (A: Kessipoughou, B: Djibilong), sand flies (C: Kessipoughou, D: Djibilong) and biting midges (E: Kessipoughou, F: Djibilong) along time.** The Shapiro-Wilk test (*alpha* level of 0.05) for all data had a p-value >0.05, indicating that data were normally distributed. Therefore, the mean values of the Shannon index *H* were compared at different time points (11 months of sampling, from May 2012 to April 2013) using one-way analysis of variance (ANOVA) with an *alpha* level of 0.05. The ANOVA revealed a significant variation of the mean diversity only for mosquitoes in Djibilong (panel b: F-value = 2.9; Df = 10; p-value = 0.01). The variation of the other mean *H* values (Diptera group and cave) was non-significant (panel a: F-value = 1.17, Df = 10, p-value = 0.34; panel c: F-value = 1.04, Df = 10, p-value = 0.44; panel d: F-value = 1.49, Df = 10, p-value = 0.18; panel e: not applicable; panel f: F-value = 0.14, Df = 10, p-value = 0.9;); * indicates a month without sampling.


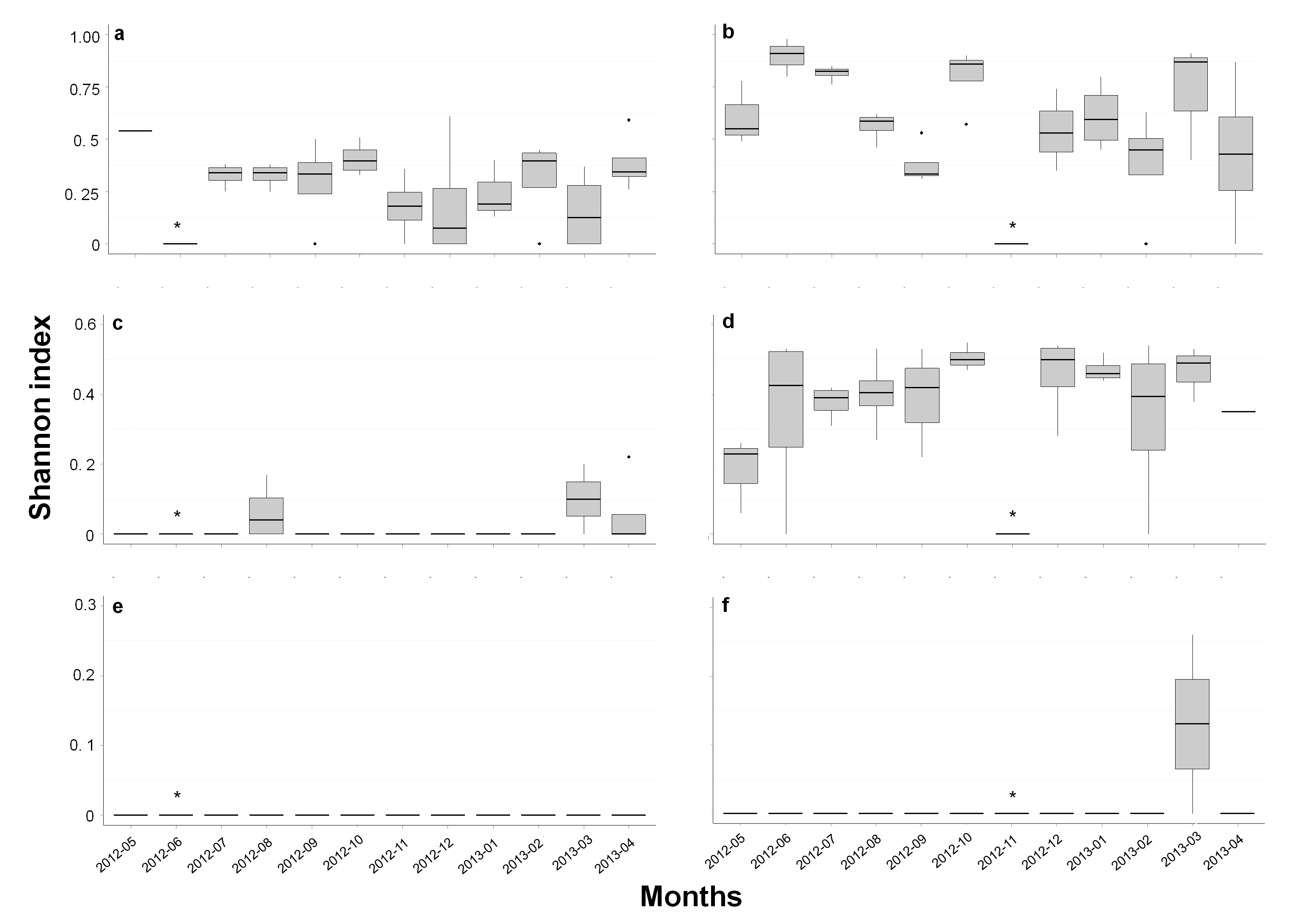

Supplement: Supplementary file 1 — Supplemental File 1 [file 41598_2017_328_MOESM1_ESM.doc]
